# Cardiac Rehabilitation Improves the QRS Fragmentation in Patients With ST Elevatıon Myocardial Infarction

**DOI:** 10.15171/jcvtr.2015.21

**Published:** 2015

**Authors:** Mustafa Bulut, Rezzan Deniz Acar, Sunay Ergün, Çetin Geçmen, Mustafa Akçakoyun

**Affiliations:** ^1^ Department of Cardiology, Kartal Kosuyolu Education and Research Hospital, Istanbul, Turkey; ^2^ Department of Physical Therapy and Rehabilitation, Kartal Kosuyolu Education and Research Hospital, Istanbul, Turkey

**Keywords:** Cardiac Rehabilitation, QRS Fragmentation, Myocardial Electrical Stability, Hypertension

## Abstract

*Introduction:* We aimed to evaluate the effect of exercise-based cardiac rehabilitation (CR) on the fragmented QRS (fQRS) in patients with ST elevation myocardial infarction (STEMI).

*Methods:* Ninety-seven patients with STEMI participated CR and 81 patients as a control group were included to the study. The trained patients were grouped according to the presence and persistence of QRS fragmentation on the electrocardiogram (ECG) before and after CR. If the fragmentation was present on the ECG at the beginning of the CR but not on the ECG at the end of CR; the transient group, if the fQRS persists after CR; the persistent fQRS group. ECGs obtained from the control group were grouped according to the presence of a fQRS on ECG.

*Results:* Among the trained patients, 45 (46%) did not have a fQRS before CR, whereas 52 (54%) presented a fQRS before CR, which was persistent in 35 patients (the persistent fQRS group) and transient in 17 patients (the transient fQRS group). Among 81 patients included in the control group, fQRS was persistent in 41 patients. Presence of fQRS on the ECG was significantly decreased with CR and it is better in trained group than the control group (P = .034). There were not significant correlations with other characteristics, except hypertension.

*Conclusion:* The existence of the fQRS decreases after CR in patients with STEMI especially in hypertensive individuals, which may be related to improved electrical stability in the myocardium as a predictor of increase in survival and decrease in major cardiac events.

## Introduction

The benefits of cardiac rehabilitation (CR) have been reported for more than 40 years. The most comprehensive data including 47 trials and reports demonstrated that the exercise-based CR improves the survival (13% for all cause mortality and 26% for cardiac mortality).^1^ Also, according to the meta-analysis of 34 randomized controlled trials by Lawler et al,^2^ CR attendees had a significantly lower risk of re-infarction than non-attendees in patients with cardiovascular diseases.The reduction in cardiovascular disease risk factors, improvement in endothelial function, improved diastolic function and positive ventricular remodelling which may provide the improvement in the electrical stability of the myocardium after a heart attack may be the responsible mechanisms for these benefits.^3-7^

However, the sudden cardiac death risk was increased in patients with myocardial infarction (MI).^8,9^ In previous studies, myocardial scarring was found associated with alteration in QRS morphology which may leads to fragmentation of QRS complex or a terminal conduction delay on the 12-lead ECG.^10,11^ A fQRS can be defined as presence of an additional R wave or indentation of the tip of the S wave or fragmentation in more than one contiguous leads for the same coronary artery region.^12^ The QRS duration should be less than 120 milliseconds because left bundle branch block may result in similar activation pattern. The different RSR’ morphologies may symbolize different conduction and depolarization models in the left ventricle which is related to myocardial scarring or fibrosis after MI. Persistent fQRS was shown as a predictor of decreased survival after ST elevation myocardial infarction (STEMI).^13^ Because fQRS is a simple and reliable tool to predict improved electrical stability of the myocardium, we aimed to evaluate the effect of exercise-based CR on fQRS in patients with STEMI.

## Materials and Methods

Ninety-seven patients with STEMI and completed phase II CR (30 exercise sessions) and exercise training program were included in the study as the training group. The control group was composed of 81 patients who had history of STEMI but could not attend or complete CR program. All patients were underwent primary PCI with the first time STEMI and also revascularized if there was an additional coronary lesion. They were medicated according to the current guidelines.^14^ Before participating the CR program, patients in the trained group underwent exercise testing by using standard Bruce protocol. Also, at the end of the CR program, they underwent to the same exercise testing protocol to evaluate the exercise capacity .

Serial 12-lead ECGs (The Cardiac Science Burdick Atria 6100 EKG Machine) were obtained before and after CR. The resting 12-lead ECG with 150 mHz was analyzed by 2 independent readers. The presence of fQRS in 2 or more adjacent anterior leads from V1 trough V6 was noted as a determiner of an anterior MI, the presence of fQRS in two or more adjacent lateral leads such as I, aVL, V5 and V6 was noted as a determiner of a lateral MI and the presence of fQRS in two or more adjacent inferior leads; II, III, and aVF was noted as a determiner of an inferior MI. If no fragmented QRS was seen on the 12-lead ECGs before CR, patients were classified as ‘the no fQRS’ group. If the present QRS fragmentation persists after CR, we classified the patients as ‘the persistent fQRS’ group. Patients were classified as ‘the transient fQRS’ group if the fragmentation was present on the ECG at the beginning of the CR program but disappeared at the end of CR. The trained group were participated in CR 2-4 weeks after STEMI and the program was lasted for 6 weeks. Therefore, we assessed the ECGs of the control group approximately 3 months after STEMI and classified the patients into ‘the persistent fQRS’ group and ‘the no fQRS’ group.

Patients who had typical bundle-branch block pattern (QRS >120 milliseconds) or incomplete right bundle-branch block were excluded from the study.

This study was performed in compliance with the Declaration of Helsinki, and also approved by local ethical committee. Each patient gave written consent before CR.

### Cardiac Rehabilitation

CR program was performed in the Cardiac Rehabilitation Department of our hospital by a multidisciplinary team consisting of physical therapy and rehabilitation specialist, cardiologist, a specialized nurse, consultant psychiatrist and dietitian. The phase 2 CR program was performed to the training group and the exercise prescription was individualized. The minimum frequency for exercising was 5 times weekly during the 6 weeks. Patients were allowed 30-60 minutes for each session. In addition to exercise, lifestyle changes were encouraged such as weight reduction and smoking cessation.

Lower-risk patients following STEMI were enrolled in this study. The factors that can increase the risk such as; elderly, left ventricular dysfunction, myocardial ischemia symptoms, skeletal disabilities, neuropathies due to diabetes autonomic dysfunction, peripheral artery disease and pulmonary diseases, were considered. High risk members with ongoing ischemia, poor exercise capacity (performing less than 5 metabolic equivalents [METs]), decrease in systolic blood pressure (BP) of 15 mm Hg or more with exercise stress test, ventricular arrhythmia, patients with pacemakers and defibrillators were excluded from the study.

### Statistics

Statistical analysis was performed using the SPSS version 15.0 software for Windows (IBM, Armonk, NY). Categorical data were expressed as frequencies. Continuous variables were presented as the mean ± standard deviation. χ2 test was applied to compare the influence of the categorical variables. The *t* test and one way analysis of variance (ANOVA) were used to test for the differences between groups. The relationship between the demographic properties of patients and an fQRS were analyzed by linear regression methods. A P value <.05 was considered to indicate statistical significance.

## Results

Baseline demographics and clinical characteristics of 97 patients for trained group and 81 patients for control group are summarized in Table 1. There were no significant differences between the groups with respect to demographic data. Among the 97 patients included in the study, 45 individuals (46%) did not have a fQRS on the 12-lead ECG (the no fQRS group) before CR, whereas 52 individuals (54%) presented a fQRS before CR. The fQRS was persistent in 35 individuals (the persistent fQRS group) and transient in 17 individuals (the transient fQRS group). However, the fQRS was present in 9 (25%) of the anterior leads, 7 (20%) of the lateral leads, and 19 (54%) of the inferior leads after CR. Among 81 patients included in the control group, the fQRS was persistent in 41 patients. The fQRS was present in 13 (32%) of the anterior leads, 8 (21%) of the lateral leads, and 20 (47%) of the inferior leads. It must be pointed out that the fragmentation was mostly found in the inferior territory in both groups. There was 97% agreement between the 2 readers in defining fQRS. The comparison of the 2 groups is given in Table 2.

**Table 1:** Patient Demographics and Clinical Characteristics

	**Trained Group (n = 97)**	**Control Group (n = 81)**	*** P *** ** Value**
Age (years –mean± SD )	58 ± 7	59 ± 8	.586
Sex (male/female)	79 male/18 female	54 male/27 female	.057
Diabetes (n)	34 (35%)	25 (30%)	.557
Hypertension (n)	52 (53%)	51 (62%)	.210
Hyperlipidemia (n)	42 (43%)	26 (32%)	.127
Smoking (n- current)	36 (37%)	28 (34%)	.726
Angina to balloon time (h)	3.8 ± 2.4	3.5 ± 2.5	.411
IRA			.126
LAD (n)	38 (40%)	35 (44%)	
CX (n)	18 (18%)	21 (26%)	
RCA (n)	41 (42%)	25 (30%)	

Abbreviations: EF: ejection fraction, CO: cardiac output, LV: left ventricle, IRA: infarct related artery, LAD: left anterior descending, CX, circumflex, RCA, right coronary artery.

**Table 2:** Baseline Characteristics According to the Electrocardiographic Signs of the Patients. Only Hypertension is Associated With fQRS

	**Trained Group – fQRS**			**Control Group – fQRS**		*** P *** ** Value**
	**Persistent** **(n = 35)**	**Transient** **(n = 17)**	**None** **(n = 45)**	**Persistent** **(n = 41)**	**None** **(n = 40)**	
Age (y)	57	59	58	61	56	0.069
Gender (male)	30	14	35	26	28	0.216
EF (%)^a^	47 ± 5	47 ± 7	49 ± 6	46 ± 6	48 ± 7	0.385
HT (n)	29	8	15	31	20	0.014
DM (n)	13	8	13	16	9	0.264
Smoking (n)	14	9	13	17	11	0.550
HPL (n)	19	6	17	14	12	0.247
IRA (n)						0.097
LAD	13	8	17	21	14	
CX	8	2	8	12	9	
RCA	14	7	20	8	17	

Abbreviations: EF: ejection fraction, HT: hypertension, DM: diabetes mellitus, HPL: hyperlipidemia, IRA: infarct related artery, LAD: left anterior descending, CX, circumflex, RCA: right coronary artery. ^a^ Mean ± SD.

**Figure 1:**
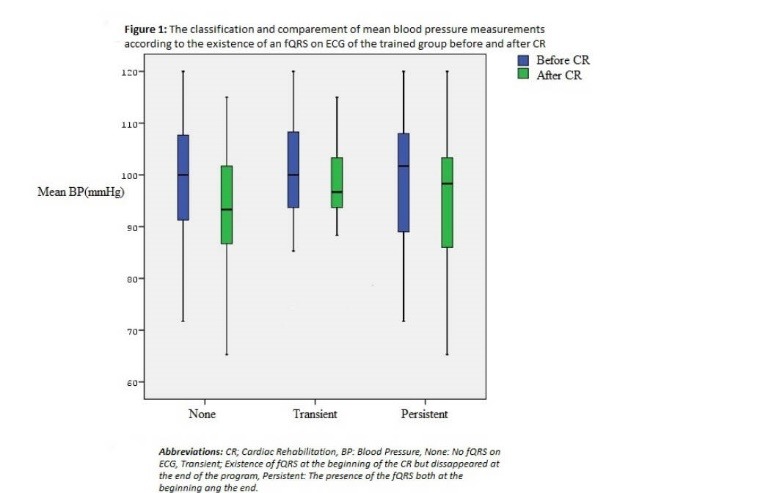
The classification and comparison of mean blood pressure measurements according to the existence of a fQRS. Abbreviations: BP, blood pressure; CR, cardiac rehabilitation; None; no fQRS on ECG; Transient, existence of fQRS at the beginning of the CR but disappeared at the end of the program; Persistent; presence of fQRS both at the beginning and the end.

**Figure 2:**
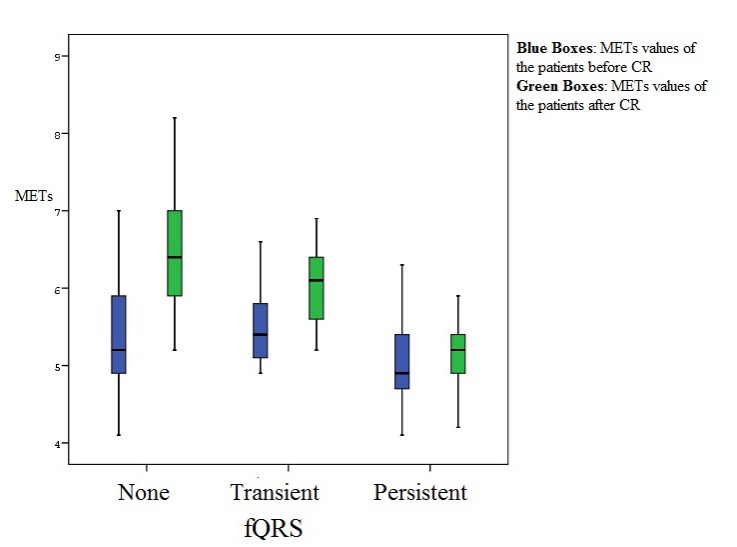
The classification and comparement of the existence capacity of the trained patients according to the existence of a fQRS on ECG after and before CR. Abbreviations: BP, blood pressure; CR, cardiac rehabilitation; None; no fQRS on ECG; Transient, existence of fQRS at the beginning of the CR but disappeared at the end of the program; Persistent; presence of fQRS both at the beginning and the end.

The fQRS existence on the ECG was significantly decreased with CR and it is better in trained group than the control group. (*P*=.034, Table 3). There was statistically significant decrease in mean BP after completion of the CR and exercise training program in trained group. The classification and the comparison of the mean BP according to the presence of a fQRS on ECG is demonstrated in Figure 1. As seen in Figure 2, there was a statistically significant increase in the measured METs after the training period in all patients with a fQRS or not. Although the left ventricular ejection fraction (EF) was increased significantly after CR (*P*=.031), there was not any statistical difference in EF between trained group and CR rehabilitation group ( *P* = .157 ). In linear regression analysis, only hypertension (β: -.185, *P* = .014) was associated with an fQRS. Also, the exercise capacity was found to be negatively correlated with the fQRS (r: - 0.69, *P *< .01). There were not any significant correlation with other characteristics such as infarct related artery (IRA, β: .155, *P* = .068), gender (β: .021, *P* = .788), age (β:.109, *P* = .152), hyperlipidemia (HPL, β:-.264, *P* = .075), diabetes (DM) (β:-.003, *P* = .998), smoking (β: 0.233, *P* =.139) and left ventricular EF (β: -.164, *P* = .87).

**Table 3:** EF and fQRS of the Patients Before and After Cardiac Rehabilitation and the Control Group

	**Trained Group (n:97)**		**Control Group (n = 81)**	*** P *** ** value**
	** Before CR**	**After CR**		
**EF (% - Mean ± SD )**	46 ± 7	48 ± 6	47 ± 6	.157
**fQRS (n)**	52	35	41	.034

Abbreviations: EF: ejection fraction.

## Discussion

In patients with acute myocardial infarction (AMI), the fQRS on the ECG recorded during the first 48 hours or 2 months later is a significant predictor of long -term mortality.^13,14^ Even in patients without structural heart disease, the presence of a fQRS was found to be associated with frequent premature ventricular contractions.^15^ Our study is the first to evaluate the effect of exercise-based CR on fQRS in patients with STEMI. The fragmented QRS was persisted in 36% of individuals after the CR. Also, our data confirms that a fQRS is mostly located in the inferior territory, which keeps in with the previous studies. In a study by Maskoun et al,^16^ infarct size measured with cardiac magnetic resonance imaging (CMR) and a fQRS existence on the 12-lead ECG were described as a predictor of major cardiac events after AMI. Because the fragmentation is linked to inhomogeneous ventricular activation due to the ischemia and scarred tissue in the myocardium, it is important to figure out if CR plays a role in the improvement in fQRS.

According to a recent study by Lorgis et al,^17^ regardless of the existence of a Q wave, transient fQRS correlates with recurrent MI, whereas the fQRS was found as a predictor of decreased survival in patients with AMI. Also, Das et al^18^ confirmed that the fQRS has a substantially higher sensitivity than the Q wave in patients with known or suspected CAD. According to their study, the fQRS has a higher negative predictive value for myocardial scar than does the Q wave. Nevertheless, later, they reported another study on fQRS and its interaction with mortality in a population of both nonischemic and ischemic cardiomyopathy patients; supporting the theory that fQRS is a risk factor for major cardiac events.^19^ The presence of a fQRS in a focal region indicates the presence of a myocardial scar in the relevant territory. In a study among patients with chronic total occlusion without prior MI, the existence of fragmented QRS was found associated with inadequate collateral coronary circulation.^20^ One of the underlying mechanism for beneficial effects of CR may be the improved coronary collateral circulation by CR.

However, the prognostic significance and the importance of the fQRS is well defined and more clearly understood in MADIT II study by Brenyo et al^21^ QRS fragmentation was demonstrated as a predictive of decreased survival in patients with ischemic cardiovascular disease and depressed left ventricular function. Beyond the effect of the fQRS on ventricular arrhythmias, the incidence of atrial fibrillation in the postoperative period was found independently related to the presence and number of a fQRS among patients undergoing CABG surgery.^22^

In comprehensive analysis of this study adjusted for the relevant confounders such as age, gender, IRA, DM, HPL and left ventricular EF; only hypertension was found to be associated with a fQRS. Onalan et al^23^ showed that fQRS presence was increased in hypertensive patients. However, the benefit with CR was seen especially in hypertensive patients in our study which may also suggests that controlling of the BP may contribute to decrease in the fQRS. Because the exercise capacity was shown negatively correlated with fQRS, the beneficial effect of exercise on BP may be responsible for this result.

## Study Limitations

It is evident that the high-risk patients benefit from CR more, therefore, the most important limitation of this study is the patients included are at low risk. We do not know how many patients in the control group had transient fQRS. Because they did not attend CR program, we do not know the early fQRS ratio on ECG. Therefore, we assume that the fQRS we have seen on the ECG was persistent in the control group. This is a retrospective study with a relatively short follow-up duration. Large scale further experimental studies are needed to explain the exact interplay between fragmentation of the QRS and survival in patients with AMI and participated CR.

## Conclusion

This study suggests that the fQRS decreases after CR independent from EF in patients with STEMI and successfully revascularized by primary PCI. Decrease in fQRS may be related to improved electrical stability in the myocardium as a predictor of increase in survival and decrease in major cardiac events. However, this benefit can be particularly seen in hypertensive individuals‏.
